# Is there any correlation between otitis media and dental malocclusion in children? A systematic review

**DOI:** 10.1007/s40368-023-00807-0

**Published:** 2023-06-20

**Authors:** E. Bardellini, F. Amadori, M. L. Garo, A. Majorana, G. Conti

**Affiliations:** 1 Department of Medical and Surgery Specialties, Radiological Sciences and Public Health, School of Pediatric Dentistry, University of Brescia, Dental Clinic, P.le Spedali Civili N.1, 25133 Brescia, Italy; 2 University of Insubria, Varese, Italy

**Keywords:** Children, Otitis, Occlusion, Malocclusion

## Abstract

**Purpose:**

This study aims to evaluate whether there is a correlation between otitis and dental malocclusions.

**Methods:**

Electronic databases were searched for observational studies published until July 2021 without language or time restrictions. PROSPERO: CRD42021270760. Observational studies on children with and without OM and/or malocclusion were included. After removing duplicates and excluding not-eligible articles, two reviewers screened relevant articles independently. Two reviewers independently extracted data and assessed data quality and validity through the Newcastle–Ottawa Scale (NOS) quality assessment tool for non-randomized studies.

**Results:**

Five studies met the selection inclusion criteria and were included in the studies for a total of 499 patients. Three studies investigated the relationship between malocclusion and otitis media, while the remaining two studies analyzed the inverse relationship and one of them considered eustachian tube dysfunction as a proxy of OM. An association between malocclusion and otitis media and vice versa emerged, although with relevant limitations.

**Conclusion:**

There is some evidence that there is an association between otitis and malocclusion; however, it is not yet possible to establish a definitive correlation.

## Introduction

Otitis media (OM) is an inflammation process of the middle ear (Klein and Bluestone1998). By the age of 3, more than 80% of children may suffer from one or more episodes of OM, with recurrences in about a third of them (Bernkopf et al. 2016). Recurrent Acute Otitis Media (RAOM) is defined in the case of three acute episodes within 6 months or four episodes during a year. RAOM is believed to depend on morphological and/or functional disturbances of the eustachian tube (ET), like variations in shape and size or in angulation, width, and length (Bernkopf et al. 2016; Klein and Bluestone 1998). As a matter of fact, RAOM is more common in pre-school aged children than in adults, due to their shorter and more horizontal ET, which subsequently tends to modified with craniofacial growth (Mann et al. 1979; Nery Cde et al. 2010; Teele et al. 1989). In addition to anatomical factors, other condition, such as adenoid hypertrophy, allergy, upper respiratory tract infections, and gastroesophageal reflux, would favor RAOM onset.

Some studies proposed the pathogenetic role of dental malocclusion as well (Schilder et al. 2017). Costen (1934) was the first who describe deep bite as a risk factor for the eustachian tube dysfunction (ETD). Afterward, several studies on the pediatric population supported the relationship between the mandibular position and the risk of OM (Bernkopf et al. 2016; De Stefano et al. 2009; Giuca et al. 2011). A recent narrative review (Bernkopf et al. 2022) collected the current evidence about the impact of temporomandibular joint (TMJ) dysfunction on the onset of OM. The anatomical and physiological relations between middle ear structures—particularly ET, TMJ, and the mandible—seem to endorse the role of the stomatognathic system in the pathogenesis of OM. The possibility of identifying a determinant factor for otitis development, which can be early corrected through orthodontic therapy, is fascinating as it would open new diagnostic and therapeutic perspectives. According to a recent meta-analysis (Lombardo et al. 2020), the worldwide prevalence of malocclusion is 56% (without differences in gender and dentition). Therefore, being the malocclusion a common condition in children, whether the association with ear infections is “casual or causal” can be questioned. As far as we know, there are no review which systematically analyze the possible correlation between otitis and dental malocclusions. This is the first study which seek to clarify the main questions about malocclusion and RAOM: what do we know about this association? and which are the malocclusions mainly associated with the recurrence of otitis media, if any association exist?

## Materials and methods

The materials and methods were based on the PRISMA (Preferred Reporting Items for Systematic Reviews and Meta-Analysis) guidelines. Prospero registration: CRD42021270760.

### Information sources and search strategy

A systematic search was carried out on PubMed, Embase, Web of Science, and Scopus from May to July 2021 without time and language restrictions.

The components of the PECO question were as follows: (Population) children aged 0–10 years; (Exposure) children with OM and/or malocclusion; (Outcome) malocclusion if OM was diagnosed first or viceversa OM if malocclusion was diagnosed first. The search strategy is reported as supplementary material.

### Study selection

The complete list of articles obtained through the systematic search was scrutinized to remove duplicates and select the potentially relevant articles based on the title to answer the research question. Subsequently, the abstract screening was performed, as well. Two reviewers independently selected the eligible studies (EB and FA). From the remaining potentially relevant articles, those that met the inclusion and exclusion criteria were selected through full-text reading. Finally, the reasons for exclusion were recorded.

Two authors (EB and FA) independently selected the subsequent article selection. When there was disagreement, a third experienced reviewer (AM) was consulted to achieve a consensus.

### Inclusion/exclusion criteria

The inclusion criteria were as follows: (i) studies including children from 0 to 10 years with OM; (ii) observational studies. Exclusion criteria were: children suffering from syndromes, sleep disorders, children with cleft-palate, children suffering from allergies, temporomandibular joint disorders, and children undergoing adenoidectomy/tonsillectomy.

### Data extraction

Two reviewers (FA and MLG) independently extracted the data from the full texts of the studies that fulfilled the inclusion criteria. Disagreements were resolved through team discussions. The primary outcome analyzed in this review was the number of children with OM and/or malocclusion.

Data extraction was organized in tables that included the following information:

1. Study characteristics: name of the first author, year, country, sample size, and study design.
2. Participant characteristics: age, gender, and cofounders.
3. Malocclusion and OM criteria definitions.
4. Primary results.

### Data synthesis

All the data from the eligible articles were synthesized into a narrative summary. In addition, the characteristics of each study, which included study characteristics, children characteristics, type of malocclusion, primary results, and conclusions, were reported.

### Quality assessment

Risk of bias of included studies was assessed using the Newcastle–Ottawa Scale (NOS) quality assessment tool for non-randomized studies (http://www.ohri.ca/programs/clinical_epidemiology/oxford.asp).

## Results

### Study selection

**Fig. 1:**
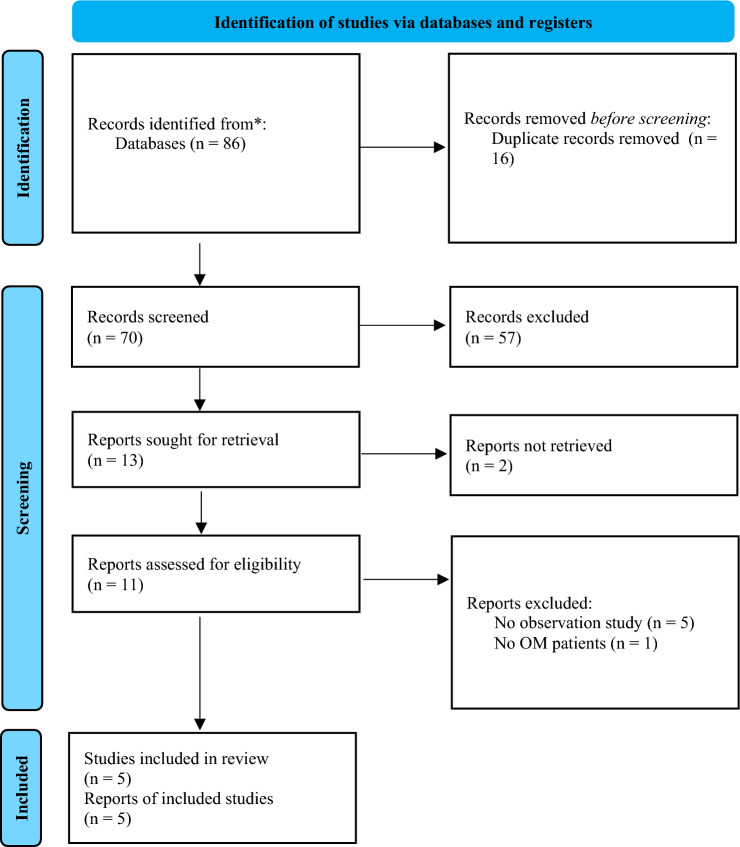
PRISMA flow-chart – Version 2020

A flow diagram of the search strategy results is presented in Fig. 1. After removing 16 duplicates, a total of 70 articles were obtained. From those 70 articles, 57 studies were excluded after reading their titles and abstracts. Finally, 13 studies were sought for retrieval. Still, only 11 were assessed for eligibility, because one report was not retrieved, and one duplicated another already included article. Of these 13 studies, five were not observational, and one had no patients with otitis media. Therefore, a total of five studies were included in the review (Table 1).

**Table 1:** Summary characteristics of included studies (*n* = 5)

Author	Country	Objective	Study design	Age	Sample size	Malocclusion or OM definition used as criteria to ascertainment	Cofounders	Sex	Evidence
Watase et al. (1998)	USA	Malocclusion in children with otitis media	Survey	< 6 years	108	Class II or Class III relationships for the primary canines on the right and left sides; distal step relationship; overbite > 70%; overjet > 5 mm; presence of an anterior open bite; presence of an anterior crossbite; presence of a posterior crossbite	Pacifier sucking habits, thumb/finger-sucking habit, mouth breathing habit, snoring, allergies, asthma, upper respiratory infections, history of breastfeeding, history of bottle feeding, history of otitis media in the family	64 males and 44 females	56/108 (51.9%) of children with malocclusion Prominent types of malocclusions: anterior open bites (17%) and overbites > 70% (17%) and accounted for about 70%% of all malocclusions No statistically significant association between all explanatory variables (factors related to otitis media) and malocclusion
McDonnell et al. (2001)	USA	Relationship between dental overbite and Eustachian Tube Dysfunction (dysfunction which predisposes to OM)	Observational study	2–6 years	105	Deep bite (70%), Overjet > 3 mm, non-mesial step occlusion	Age, medical/environmental history, family history of OM, Non-breast-fed (0–6 mo), URI > 5 per year, home cigarette smoke exposure, Tonsillectomy, Snoring history, Seasonal allergy history, Pacifier History	NR	57% (*n* = 60) of the children had ETD Deep bite > 70% in 32% (*n* = 34) of children Overjet > 3 mm in 23% (*n* = 24) of children Mesial step terminal plane relationship in 80% (*n* = 84) of children Flush terminal plane in 15% (*n* = 16) of children Distal step relationship in 5% (*n* = 5) of children Univariate analysis: Children with ETD were approximately twice as likely to have a deep bite as children without ETD (*P* = 0.02) Children with a deep bite were significantly more likely to be white, to have a history of seasonal allergy, and to have a non-mesial step occlusion Children with a deep bite did not have an open bite Children with ETD were significantly more likely to have a family history of middle ear disease, to have five or more URIs per year, and to be under three years Multivariate logistic regression: Children with deep bites were 3.5 times more likely to have ETD than children without (95% confidence interval [CI]: 1.1, 11.2; P = .03) Children with deep bites were 2.8 times more likely to have ETD greater (95% CI: 1.1, 7.1; *P* = .03) than children without. Other independent risk factors for ETD in the final model were family history of OM (odds ratio [OR] = 3.6; 95% CI: 1.3, 9.8; *P* = .01) and age < 3 years (OR = 5.8; 95% CI: 1.2, 29.2; *P* = .3)
Kim et al. (2008)	USA	Relationship between dental OM and the anatomic form of the hard palate	Retrospective study	4–6 years	175	Palatal vault form: Class I—medium—(palatal slope forms an angle of 30 to 45 degrees to the horizontal plane; the palatal slope is characterized by a round curvature that forms a slight concavity); Class II—high/steep—(palatal slope is steeper compared to that of the medium plate; an angle greater than 45 degrees; the slope in usually round and pronounced (convex) at the coronal third); Class III—low/flat—(shallow palate; the palatal slope is flatter compared to that of the medium palate; an angle less than 30 degrees is created by the previously described landmarks; the palatal slope is usually short, and the mid-palate has a flat surface)	Age, race, gender, systemic health, tobacco smoke exposure, method of feeding during infancy, history of intubation, history of a finger-sucking habit and pacifier use, history of acute OM, age during the initial episode of acute OM, number of episodes of acute OM experienced, treatments rendered to manage episodes of acute Om	NR	148 (85%) of the total sample had a positive history of AOM, with 76% experiencing AOM before age 1 and 61% experienced more than three bouts of AOM High palatal vault was a significant finding in children that experienced AOM before age 1 (OR: 3.49; 95%CI: 1.14, 10.69; *P* = 0.03) Children with high palatal vaults underwent myringotomy and tympanostomy tube placement procedures more often than the rest of the study population (OR: 2.49; 95%CI: 1.15,5–39; *P* = 0.02)
Giuca et al. (2011)	Italy	Correlation between OM and dental malocclusion	Case–Control	Study group: 7.7 ± 0.9 years; Control group: 7.9 ± 1.1 years	50 (25 study group; 25 healthy subjects)	OM and Malocclusion variables were defined, but criteria were not identified	NA	26 males (13 in the study group and 13 in the control group) and 24 females (12 in the study group and 12 in the control group)	Bilateral posterior crossbite and unilateral posterior crossbite were more observed in study group (11/44% and 8/32%) than in control group (4/16% and 0/0%) with a statistically significant difference (*P* = 0.046 and *P* = 0.026)
Bernkopf et al. (2016)	Italy	The role of dental malocclusion treatment in the outcomes of RAOM	Case–Control	Study group: 6.6 ± 1.9 Control group: 5.3 ± 1.9	61 consecutive children	3 AOM episodes within 6 months, or four episodes during one year—Malocclusion: sagittal discrepancies (increased overjet or anterior crossbite); vertical discrepancies (open bite or deep bite); transversal discrepancies (deviated bite or unilateral crossbite)	Age, Skin-prick test (negative vs positive), adenoid hypertrophy, number of AOM recurrences	35 females and 26 males	Children in group A treated for dental malocclusion were strongly associated with a lower number of acute episode recurrences at both univariate (OR: 37, 9%CI: 7.18–233.0; p < 0.0001) and multivariate analysis (*P* = 0.0001)

AOM: Acute Otitis Media—OM: Otitis Media—RAOM: Recurrent Acute Otitis Media

### Risk of bias

The overall risk-of-bias assessment of the included studies is presented in Table 2. We judged all studies at low risk of bias, because they performed an appropriate method of patients’ selection. For comparability, only a study (Giuca et al. 2011) was judged at high risk of bias, because possible cofounders were not reported. In addition, all studies were evaluated at a low risk of bias given an appropriate ascertainment of exposure (medical charts) and the use of the same method of ascertainment for patients with and without OM and/or malocclusion.

**Table 2:** Risk of bias—Newcastle–Ottawa Scale (NOS) quality assessment tool for non-randomized studies

	Selection	Comparability	Exposure
Watase et al. (1998)	****	**	***
McDonnell et al. (2001)	****	**	**
Kim et al. (2008)	****	**	***
Giuca et al. (2011)	****	NR	**
Bernkopf et al. (2016)	****	**	**

### Study characteristics

We provided a descriptive summary of the information on study design, participants, malocclusion, and OM in Table 1. We included a total of five studies: one survey (Watase et al. 1998) and four observational studies (Bernkopf et al. 2016; Giuca et al. 2011; McDonnell et al. 2001; Kim et al. 2008). Reports were published between 1998 and 2016. Three studies were conducted in USA (Kim et al. 2008; McDonnell et al. 2001; Watase et al. 1998) and two in Italy (Bernkopf et al. 2016; Giuca et al. 2011). Three studies included more than 100 participants (Kim et al. 2008; McDonnell et al. 2001; Watase et al. 1998), while the remaining other studies had 50 (25 per group) and 61 (group A: 32; group B: 29) participants, respectively.

Three studies investigated the relationship between otitis media and malocclusion (Bernkopf et al. 2016; Giuca et al. 2011; Watase et al. 1998), while the remaining two studies investigated the relationship between possible malocclusion and otitis media (Kim et al. 2008) and eustachian tube dysfunction (McDonnell et al. 2001).

Participants were recruited from pediatric dental clinics (Kim et al. 2008; Watase et al. 1998), departments of otolaryngology (Giuca et al. 2011; McDonnell et al. 2001), and the otorhinolaryngology unit (Bernkopf et al. 2016).

The five studies included a total of 499 participants. The median sample size was 105 (range 50 to 175). Three studies included children aged less than 6 years (Kim et al. 2008; McDonnell et al. 2001; Watase et al. 1998); the other two studies included children with a mean age of 5.3 ± 1.9 years (Bernkopf et al. 2016) to 7.9 ± 1.1 (Giuca et al. 2011). Sex was not reported in all included studies. Malocclusion was defined differently: Watase et al. (1998) defined malocclusion as class II or class III relationships for the primary canines on the right and left side, overbite > 70%, overjet > 5 mm, presence of an anterior open bite, presence of an anterior crossbite, and presence of a posterior crossbite. McDonnell et al. (2001) identified malocclusion as deep bite > 70%, overjet > 3 mm, and non-mesial step occlusion. Kim et al. (2008) identified palatal value-form classifying participants according to Class I (palatal slope forms an angle of 45° to the horizontal plane and is characterized by a round curvature that forms a slight concavity), Class II [palatal slope is steeper compared to that of the medium plate, forms an angle greater than 45° and is round and pronounced (convex) at the coronal third], and Class III (shallow palate in which the palatal slope is flatter compared to that of the medium palate and forms an angle less than 30°; the palatal slope is short and the mid-palate has a flat surface). Malocclusion was also defined as sagittal discrepancies (increased overjet or anterior crossbite), vertical discrepancies (open bite or deep bite), or transversal discrepancies (deviated bite or unilateral crossbite) (Bernkopf et al. 2016; Giuca et al. 2011). Otitis media was defined as the recurrence of three episodes within 6 months or four episodes in 1 year (Bernkopf et al. 2016; Giuca et al. 2011). Four studies out of five reported cofounders as pacifier history, medical/environmental history, family history of OM, home cigarette smoke exposure, age, systemic health, and finger-sucking habits (Bernkopf et al. 2016; Kim et al. 2008; McDonnell et al. 2001; Watase et al. 1998), and one studied the possible mediation of age, skin-prin test, adenoid hypertrophy, and the number of acute otitis media recurrences (Bernkopf et al. 2016), while only one study did not take into account possible cofounders (Giuca et al. 2011).

### Prevalence of malocclusion in patients with otitis media

Three studies investigated the prevalence of malocclusion in patients with otitis media. In a survey (Watase et al. 1998) carried out on 108 children aged less than 6 years and diagnosed with OM, a prevalence of malocclusion of 51.2% (56/108) was observed, predominantly anterior open bite and an overbite measuring greater than 70%. A logistic regression analysis showed no significant association between OM and malocclusion.

Giuca et al. (2011) observed a higher prevalence of posterior crossbite and unilateral posterior crossbite in patients with otitis media than in healthy subjects (44% and 32% in the study group vs 4% and 0% in the control group, *p* = 0.046 and *p* = 0.026). Finally, Bernkopf et al. (2016) showed that children with OM treated to reduce dental malocclusion were less subjected to acute episode recurrences at both univariate (OR 37, 95%CI: 7.18,233.0, *p* < 0.0001) and multivariate analyses (*p* = 0.0001).

### Prevalence of otitis media in patients with malocclusion

Only two studies (Kim et al. 2008; McDonnell et al. 2001) analyzed the prevalence of OM in patients with malocclusion. In particular, one of these studies (McDonnell et al. 2001) did not directly investigate the relationship between these two pathologies but reported findings of the association between malocclusion and eustachian tube dysfunction, considering this last dysfunction as a precursor of otitis media. In a sample of 105 children aged less than 6 years, McDonnell et al. (2001) showed that children with deep bites were 2.8 times more likely to have ETD than children without ETD. Kim et al. (2008) analyzed the relationship between the anatomic form of the hard palate and the prevalence of otitis media in a sample of 175 patients aged 4–6 years: they found that high palatal vault was more observed in children who had experienced acute media otitis before age 1 (OR: 3.49, 95%CI: 1.14, 10.69, *p* = 0.03).

## Discussion

This review aimed to investigate whether exists a correlation between otitis and malocclusion. Among malocclusions, the deep bite resulted significantly associated with ETD, especially under the age of 3 (Kim et al. 2008; McDonnell et al. 2001; Monsell and Harley 1996). As concerns this association, it has been speculated that a deep dental overbite may affect the temporomandibular joint (TMJ) or the tensor veli palatini muscle. As for the TMJ, a deep bite can induce a distal displacement of the condyle, which causes pressure and inflammation in the retro‐discal tissue and TMJ capsule, spreading through the petrotympanic fissure directly to the ET; this inflammation may decrease the ventilation of the middle ear and mastoid spaces, favoring the onset of OM (Sicher 1948; Stack and Funt 1997). As for the tensor veli palatini, Costen (1934) postulated that the deep bite causes a contraction of the tensor palatini muscle, which is the only muscle that acts directly on the ET. Therefore, disturbance in the normal function of this muscle might prevent proper ventilation of the middle ear and mastoid space and predispose to OM. Other authors suggested changes in the angulation of the tensor palatini muscle in children with small nasopharyngeal anatomy, which may result in an altered vector of force being applied to the ET during opening through contraction of this muscle and in a decreased efficiency of middle ear ventilation (Klein and Bluestone 1998). Bernkopf et al. (2016) showed that children treated to reduce deep bite were less prone to acute episode recurrences, confirming the correlation between deep bite and otitis. In the previous years, Watase et al. (1998) denied the association between malocclusion and OM, but they did not examine individual malocclusion variables and did not have a control group, making the results difficult to interpret. Studies having control groups reported interesting anatomical relations. According to Joseph et al. (1998), children with class II malocclusions had smaller nasopharyngeal dimensions than those with other occlusal classes, in which there was no reduction of the vertical dimension. Di Francesco et al. (2008) found that children with OM presented differences in the facial anatomical features, especially facial depth and facial axis were found to be greater in the OM group than in the control group, similarly to what happens in the case of deep bite (Kitajiri et al. 1985; Mann et al. 1979). Gremba et al. (2016) observed alterations in the craniofacial morphology in children with RAOM, i.e., a posterior displacement of the palate, of the middle cranial base and of the external acoustic meatus, consistently to what happens in case of deep bite. According to these studies, therefore, there seems to be a similar anatomical conformation in patients with deep bite and in patients with otitis.

Besides deep bite, posterior crossbite resulted associated with OM (Giuca et al. 2011). Anatomically, a reduction in the transverse palatal diameter and a high palatal vault could affect the aeration mechanisms of the middle ear and decrease the tube function. Crossbite can also be associated with mandibular deviation and low lingual position, affecting the muscle groups responsible for opening the tube. The tensor veli palatini open-closed dynamics, when spastic or weakened by the nearby pterygoid muscle, play a crucial role in OM onset. After maxillary expansion, the tensor veli palatini muscle is stretched and its contractile activity improves resulting in more effective force vectoring (Bernkopf et al 2022).

One of the major limits of this review was that the definition of the two parameters to be correlated was not homogeneous in the literature. The term “malocclusion” could be intended as a class II or class III Angle relationship and/or a sagittal discrepancy (increased overjet or anterior crossbite) (Giuca et al. 2011; Nery Cde et al. 2010) and/or a vertical discrepancy (open bite or deep bite) (Bernkopf et al. 2016; Giuca et al. 2011; Nery Cde et al. 2010) and/or a transversal discrepancy (deviated bite or unilateral crossbite) (Bernkopf et al. 2016; De Stefano et al. 2009; Giuca et al. 2011; Nery Cde et al. 2010). Similarly, the term “otitis media” could refer either to the number of recurring episodes (Bernkopf et al. 2016; Giuca et al. 2011) or to the presence of an ETD (De Stefano et al. 2009; McDonnell et al. 2001; Nery Cde et al. 2010). Due to the paucity and heterogeneity of the studies on this topic, we could not narrow down the inclusion criteria to a single definition of “otitis” and “malocclusion”. We therefore extrapolated the single types of malocclusion from the included studies and discussed the findings.

Carrying the scientific evidence of the association between otitis and malocclusion would allow a multidisciplinary approach to the treatment of otitis, recognizing the role of the pediatric dentist in identifying possible risk factors for chronicity. This would also lead to develop future guidelines that can foster collaboration between otorhinolaryngologists and pediatric dentists.

## Conclusion

Considering any limitation of the present study, it has been shown that:

- there is some evidence that there is an association between otitis and two malocclusions (i.e., deep bite and crossbite), but at the moment, it is not yet possible to establish a definitive correlation, given the small number of studies and the relevant limitations, such as the lack of uniformity in definitions and data collection, variable outcomes, and non-standardized treatments.
- there is an undeniable anatomical and functional relation among middle ear, TMJ, and the mandible, for their proximity and direct and indirect connections.
- randomized-controlled trials, with homogeneous groups for confounding factors, are encouraged to establish the role of malocclusion in the onset of OM and the effectiveness of orthodontic treatment in OM prevention.

## Data Availability

All relevant data are within the paper.
